# Defining a Dichotomous Indicator for Population-Level Assessment of Dietary Diversity Among Pregnant Adolescent Girls and Women: A Secondary Analysis of Quantitative 24-h Recalls from Rural Settings in Bangladesh, Burkina Faso, India, and Nepal

**DOI:** 10.1016/j.cdnut.2023.102053

**Published:** 2023-11-30

**Authors:** Eric O Verger, Sabrina Eymard-Duvernay, Dang Bahya-Batinda, Giles T. Hanley-Cook, Alemayehu Argaw, Elodie Becquey, Loty Diop, Aulo Gelli, Helen Harris-Fry, Shivani Kachwaha, Sunny S Kim, Phuong Hong Nguyen, Naomi M Saville, Lan Mai Tran, Rock R Zagré, Edwige Landais, Mathilde Savy, Yves Martin-Prevel, Carl Lachat

**Affiliations:** 1 MoISA, Univ Montpellier, CIRAD, CIHEAM-IAMM, INRAE, Institut Agro, IRD, Montpellier, France; 2 Department of Food Technology, Safety and Health, Faculty of Bioscience Engineering, Ghent University, Ghent, Belgium; 3 Department of Population and Family Health, Institute of Health, Jimma University, Jimma, Ethiopia; 4 International Food Policy Research Institute (IFPRI), Dakar, Senegal; 5 International Food Policy Research Institute (IFPRI), Washington, DC, United States; 6 Department of Population Health, London School of Hygiene and Tropical Medicine, London, United Kingdom; 7 Johns Hopkins University, Baltimore, MD, United States; 8 UCL Institute for Global Health, London, United Kingdom; 9 Emory University, Atlanta, GA, United States

**Keywords:** dietary diversity, indicator, micronutrient adequacy, minimum dietary diversity for women, pregnant, resource-poor settings

## Abstract

**Background:**

The Minimum Dietary Diversity for Women of Reproductive Age (MDD-W) indicator was validated as a proxy of micronutrient adequacy among nonpregnant women in low- and middle-income countries (LMICs). At that time, indeed, there was insufficient data to validate the indicator among pregnant women, who face higher micronutrient requirements.

**Objective:**

This study aimed to validate a minimum food group consumption threshold, out of the 10 food groups used to construct MDD-W, to be used as a population-level indicator of higher micronutrient adequacy among pregnant women aged 15–49 y in LMICs.

**Methods:**

We used secondary quantitative 24-h recall data from 6 surveys in 4 LMICs (Bangladesh, Burkina Faso, India, and Nepal, total *n* = 4909). We computed the 10-food group Women's Dietary Diversity Score (WDDS-10) and calculated the mean probability of adequacy (MPA) of 11 micronutrients. Linear regression models were fitted to assess the associations between WDDS-10 and MPA. Sensitivity, specificity, and proportion of individuals correctly classified were used to assess the performance of MDD-W in predicting an MPA of >0.60.

**Results:**

In the pooled sample, median values (interquartile range) of WDDS-10 and MPA were 3 (1) and 0.20 (0.34), respectively, whereas the proportion of pregnant women with an MPA of >0.60 was 9.6%. The WDDS-10 was significantly positively associated with MPA in each survey. Although the acceptable food group consumption threshold varied between 4 and 6 food groups across surveys, the threshold of 5 showed the highest performance in the pooled sample with good sensitivity (62%), very good specificity (81%), and percentage of correctly classified individuals (79%).

**Conclusions:**

The WDDS-10 is a good predictor of dietary micronutrient adequacy among pregnant women aged 15–49 y in LMICs. Moreover, the threshold of 5 or more food groups for the MDD-W indicator may be extended to all women of reproductive age, regardless of their physiologic status.

## Introduction

Micronutrients are essential vitamins and minerals whose subclinical deficiencies contribute to an increased risk of morbidity and mortality [1]. A recent analysis suggested that two-thirds of nonpregnant women of reproductive age have one or more micronutrient deficiencies worldwide, with higher prevalence in low- and middle-income countries (LMICs) [2]. There are important changes in dietary requirements driven by physiologic processes during pregnancy, including increased requirements for folate, iron, vitamins B12 and B6, and zinc [3]. These deficiencies are exacerbated during pregnancy due to an additional demand for nutrients to support both fetal growth and development and maternal metabolism [4], and can result in adverse outcomes of pregnancy and birth [5], as well as maternal depression and cognitive impairment [6].

Dietary diversification is a food-based strategy that has been widely promoted to address micronutrient deficiencies [7]. To help achieve healthy diets, eating a diversity of foods is needed to help achieve healthy diets [8,9] as recommended by most dietary guidelines [10]. As a result, a large range of interventions and programs to improve nutrition through dietary diversification have been developed and has subsequently triggered a demand for a set of harmonized indicators to monitor progress. Subsequently, several simple indicators assessing dietary diversity were developed, primarily for use in global and national monitoring, and in survey contexts where more detailed dietary methods that include estimation of food quantities are not possible.

In this context, the Women’s Dietary Diversity Project developed and validated simple food group indicators with consistent and relevant meaning across different contexts and over time. The most recent example is the Minimum Dietary Diversity for Women of Reproductive Age (MDD-W), a simple population-level dichotomous indicator expressed as the proportion of nonpregnant women of reproductive age who consumed at least 5 out of 10 defined food groups over the previous 24 h [11]. MDD-W was validated using 9 data sets from 6 distinct LMICs as a proxy for a minimally acceptable level of intake adequacy of 11 micronutrients among nonpregnant women of reproductive age [12,13].

Although the initial MDD-W validation study was able to assess the performance of the indicator for nonpregnant nonlactating women and nonpregnant lactating women, this was not possible for pregnant women due to the lack of data. Recent studies have used the threshold of 5 or more food groups to determine whether pregnant women had more adequate micronutrient intakes but without further validation of this dichotomous indicator in this population group [[14], [15], [16]]. However, pregnant women generally have higher micronutrient requirements than nonpregnant women [3], which may change the performance of food group indicators in predicting adequate micronutrient adequacy in this specific population. The only validation study among pregnant women we are aware of showed that an adapted 6 or more food group threshold markedly improved the performance of the indicator in predicting micronutrient adequacy among pregnant girls and pregnant women in Bangladesh [17]. Using secondary quantitative 24-h recall data from 6 surveys in 4 LMICs, this study aimed to validate a minimum food group consumption threshold, out of the 10 food groups used to construct MDD-W, to be used as a population-level indicator of higher micronutrient adequacy among pregnant women aged 15–49 y in LMICs. We followed the methods used by previous studies on the development and validation of MDD-W to ensure comparability of the analysis and facilitate the interpretation of findings [12,13].

## Methods

### Selection of surveys

This study was based on a preidentified set of data sets that was completed by a systematic review of studies that collected dietary intakes from pregnant adolescent girls and women in LMICs, using 1 or multiple 24-h dietary recalls. Inclusion criteria were as follows: *1)* food consumption data collected among pregnant women (15–49 y) in LMICs; *2)* quantitative dietary intake data collected through 1 or multiple 24-h dietary recalls; *3)* use of relevant local food composition data with information on the 11 micronutrients included in the initial development and validation of MDD-W (vitamin A expressed in retinol activity equivalents, thiamin, riboflavin, niacin, vitamin B6, folate, vitamin B12, vitamin C, calcium, iron, and zinc); *4)* minimum sample size of 100 pregnant adolescent girls and women; and *5)* repeated 24-h dietary recalls from ≥10% of the study sample or being able to be matched with a relevant dietary intake survey with 2 nonconsecutive days of recall to estimate external within-person variance.

### Study design and participants

Six data sets with quantitative 24-h recall data collected from rural areas in Bangladesh in 2015 [17]; Burkina Faso in 2017/2019/2020 (BF1 [18]), 2020 (BF2 [19]), and 2019/2021 (BF3 [20]);, India in 2019 [21]; and Nepal in 2015 [22] were selected for analysis. Each data set is described in more detail in Supplemental Table 1, which includes their selection process. Briefly, there were 5 preidentified data sets (Bangladesh, BF1, BF2, BF3, and India) and we undertook a literature research to identify others, leading to add the data set from Nepal. The primary objectives of the included studies were to assess the feasibility and impact of maternal nutrition packages or integrated agriculture–nutrition interventions (Bangladesh, BF1, BF3, and India), to assess the efficacy of fortified balanced energy–protein supplementation (BF2), or to characterize the status and determinants of intrahousehold food and nutrient allocation, and test the effect of pregnancy interventions upon dietary intake (Nepal). None of the study samples was nationally representative. Data quality control was carried out by the data providers, including the exclusion of outliers. The representativeness of each sample has been discussed in the original articles, and primary study protocols for all sites were approved by ethical review committees (Comite d’éthique pour la Recherche en Santé MS/MRSI and Ethics Committee of Centre Muraz in Burkina Faso, Ethics Committee of Ghent University Hospital in Belgium, Suraksha Independent Ethics Committee in India, Nepal Health Research, University College London Ethical Review Committee) or institutional review boards (BRAC University in Bangladesh and the International Food Policy Research Institute, Washington, DC) [[17], [18], [19],[21], [22], [23], [24], [25]].

### Dietary data collection

In all studies, dietary data were collected using 1–3 quantitative multiple-pass 24-h dietary recalls conducted by enumerators specially trained for this purpose [26]. Participants were asked to describe all foods and beverages consumed during the preceding 24 h. Recipes were usually collected from the household member who was responsible for cooking. Portion sizes were estimated using methods best suited to local foods and contexts (e.g., previously distributed plates and bowls, common household measures, water volume, rice, images, and clay or wooden models). Only 2 data sets had repeated 24-h dietary recalls on nonconsecutive days, with 2 recalls for 19% of the sample (BF1) and 3 recalls for 87% of the sample (Nepal). Dietary data were converted into nutrient intakes using country-specific food composition tables; the application of yields and nutrient retention factors was done by data providers according to their practice, and information is available from original studies [[17], [18], [19],[21], [22], [23]].

### Minimum dietary diversity for women and 0-food group Women's Dietary Diversity Score

Among the various indicators with different food groupings developed and tested as part of the Women’s Dietary Diversity Projects I and II, the dichotomous MDD-W indicator has been shown to have a strong relationship to micronutrient adequacy and high consistency in terms of threshold, which best discriminated higher compared with lower micronutrient adequacy across various countries [12,27]. The MDD-W was constructed considering 10 mutually exclusive food groups consisting of the following: *1)* starchy staple foods, *2)* pulses, *3)* nuts and seeds, *4)* dairy products, *5)* flesh foods, *6)* eggs, *7)* dark green leafy vegetables, *8)* vitamin A-rich fruits and vegetables, *9)* other vegetables, and *10)* other fruits. The 10 food groups are summed into a score (10-food group Women's Dietary Diversity Score [WDDS-10]) ranging from 0 to 10, starting with a score of 0 and adding 1 point per food group consumed if the total consumption of the foods in the food group was ≥15 g/d (although the FAO MDD-W guidelines are to apply the 15 g limit to each food, we have decided here to stick to the methodology used for MDD-W validation for the sake of comparability). The WDDS-10 was computed using a single-day recall (the first day in case of repeated recalls). MDD-W was coded as 1 if WDDS-10 reached 5 food groups or more, and 0 if 4 or lower.

### Micronutrient requirements, usual intakes, and probability of adequacy

We used the estimated average requirements (EAR) and coefficients of variations proposed by Nguyen et al. [17], which are based on the information from the WHO/FAO [28],the National Academy of Medicine (formerly the Institute of Medicine) [29,30] and the International Zinc Nutrition Consultative Group (IZiNCG) [31]. These requirements were used regardless of the pregnancy trimester, age, or country context of the participants (Supplemental Table 2). These requirements were chosen rather than those proposed by Allen et al. [32] to enhance comparability and facilitate interpretation of findings with previous studies on the development and validation of MDD-W [12,13,17].

Analogous to previous studies on the development and validation of MDD-W [12,13], we used the probability approach to estimate the micronutrient adequacies of each of the 11 micronutrients [33]. This approach is based on information or assumption about both the distribution of nutrient requirements in the population and the day-to-day variations (within-person) of nutrient intakes. We applied a Box–Cox transformation to the nutrient intake distribution of every micronutrient to obtain normal distributions. For each participant and micronutrient in each separate data set, we calculated the best linear unbiased predictor of the individual’s usual intake [34], which was then used to calculate the probability of adequacy for every micronutrient (see Supplemental Methods). All usual nutrient intakes have been calculated solely on the basis of food intakes, excluding intakes from food supplements (e.g., fortified balanced energy–protein supplementation in BF2). When data sets contained repeated 24-h dietary recalls, the within-person variance was defined as the mean of squared intraindividual SDs. When data sets contained only one 24-h dietary recall, we used an external within-person variance estimate from a relevant dietary intake survey with 2 nonconsecutive days of recall [35,36]. We used the external within-person variance to between-person variance ratio multiplied by the between-person variance of our data set as the within-person variance in the best linear unbiased predictor calculations. A relevant dietary intake survey was defined as a survey conducted in the same geographic and seasonal context among pregnant adolescent girls or women. For Bangladesh, we used the within-person variance estimate from a subsample of the baseline study (∼20%) that also participated in the endline study conducted a year later [37]. For BF2 and BF3, we used the within-person variance estimate from BF1 because these 3 surveys were conducted in the same context (Boucle du Mouhoun, Centre-Ouest, and Haut-Bassins for BF1, Haut-Bassins for BF2, and Boucle du Mouhoun for BF3) among pregnant adolescent girls and women. For India, we used the within-person variance estimate from repeated 24-h dietary recall used to validate a Food Frequency Questionnaire among pregnant women living with or without HIV in Pune, India [38].

Probability of adequacy (PA) was calculated as the probability that a woman’s usual intake was at or above the EAR during pregnancy [33]. For each individual, we averaged the mean of the individual PAs for the 11 micronutrients to form the mean probability of adequacy (MPA). Similar to individual PAs, the MPA has a possible range of 0–1.

### Data analysis

Data were analyzed using Stata 17 (Statacorp) and the Stata syntax that was used for MDD-W validation in nonpregnant women [12,13], with a few minor revisions to match the aims of our analyses. Descriptive statistics are reported as medians (interquartile ranges [IQRs]) due to skewness of the distributions, except for age, height, weight, and energy intake, which are reported as means (SDs). Associations between the WDDS-10 and MPA (with or without adjustment for total energy intake) were assessed by fitting simple linear regressions. For the pooled sample, a mixed-effects regression model was used to examine the association between WDDS-10 and MPA, with random effect at data set level to take into account the within-survey correlation. The MPA variable was previously transformed by Box–Cox transformation for all the regression models.

We used receiver operating characteristic analysis and AUC to assess the diagnostic performance of WDDS-10 in predicting an MPA > 0.60, with an AUC of >0.70 deemed acceptable for predictive capacity. We estimated sensitivity, specificity, and percentage of correct classifications for MDD-W across data sets and in a pooled analysis. The MPA level of 0.60, as well as the interpretation thresholds, were selected to ensure comparability with the previous analysis used to validate the MDD-W [12,13]. Sensitivity (i.e., ability to correctly detect a person with an MPA > 0.60) is defined by the ratio between the true positives and the sum of true positives and false negatives. Specificity (i.e., ability to correctly detect a person with an MPA ≤ 0.60) is defined by the ratio between the true negatives and the sum of true negatives and false positives. A threshold was considered good when both sensitivity and specificity were >0.60, and it was considered fair enough if only one test characteristic was >0.60 and the other >0.50. Moreover, although we looked for the best balance between sensitivity and specificity, we favored specificity over sensitivity when trade-offs must be made, to be certain to identify the highest proportion of participants with an MPA ≤ 0.60. The percentage of correct classifications is defined by the ratio between the sum of true positives and true negatives and the sum of true positives, false positives, true negatives, and false negatives. A threshold was considered as good when the percentage of individuals correctly classified was >0.70, and it was considered fair enough if >0.60.

To understand the implications of some methodologic choices, we conducted additional robustness analyses to estimate sensitivity, specificity, and the percentage of correct classifications for MDD-W across data sets and in a pooled analysis according to 3 distinct scenarios. In the first robustness analysis, we tested 3 scenarios (Sc1, Sc2, and Sc3) where only 1 of the 3 Burkinabé data sets was included in the pooled analysis (BF1, BF2, and BF3, respectively), to consider the potentially redundant nature of using 3 surveys from Burkina Faso. In the second robustness analysis, we used the same recommendations from WHO/FAO [28], the National Academy of Medicine [29,30], and the IZiNCG [31] but considered pregnancy trimester, age, and level of bioavailability of iron and zinc (see Supplemental Table 3). In the third robustness analysis, we used the requirements proposed by Allen et al. [32], which consider age and level of bioavailability of iron and zinc but not the pregnancy trimester (see Supplemental Table 4).

## Results

### Characteristics of participants

Data were available for 4909 pregnant adolescent girls and women (Table 1), with sample sizes of the data sets ranging from 452 (BF1) to 1912 (BF3). The mean (SD) age of participants was 25.7 (6.2) y, with participants from Nepal being on average younger than pregnant women from other countries. The inclusion of adolescent girls (15–18 y) across studies varied from none (India) to ≤26% (Bangladesh), and was 7.1% in the pooled sample. The pregnancy trimester distribution was highly variable across data sets, with a near-even distribution in BF1, whereas almost all participants were in their third trimester in Nepal. Participants in their third trimester represented almost 60% of the pooled sample. Pregnant women in the Burkinabé data sets were on average taller and heavier than participants from other countries.

**TABLE 1:** Characteristics of pregnant women

Data set	*N*	Repeated recall, *n* (%)^1^	Mean (SD) age, y	Adolescent, *n* (%)	First trimester, *n* (%)	Second trimester, *n* (%)	Third trimester, *n* (%)	Mean (SD) height, m	Mean (SD) weight, kg	Median (IQR) WDDS-10
Bangladesh	598	0 (0.0)	24.0 (5.6)	160 (26.0)	0 (0.0)	328 (54.8)	270 (45.2)	1.50 (0.06)	50.3 (8.1)	5 (2)
BF1	452	84 (18.6)	29.6 (5.3)	1 (0.2)	124 (27.4)	173 (38.3)	155 (34.3)	1.61 (0.07)	59.1 (8.0)	3 (2)
BF2	470	0 (0.0)	25.4 (6.4)	37 (7.9)	16 (3.4)	188 (40.0)	266 (56.6)	1.63 (0.06)	58.9 (8.7)	3 (2)
BF3^2^	1912	0 (0.0)	27.5 (6.6)	64 (3.4)	279 (14.7)	828 (43.8)	785 (41.5)	1.63 (0.01)	61.8 (2.5)	3 (2)
India	674	0 (0.0)	25.0 (4.0)	0 (0.0)	0 (0.0)	198 (29.4)	476 (70.6)	1.50 (0.06)	51.0 (8.5)	4 (2)
Nepal	803	745 (92.8)	21.5 (3.8)	88 (11.0)	0 (0.0)	1 (0.1)	802 (99.9)	1.51 (0.05)	52.1 (6.5)	4 (1)
Pooled^2^	4909	N/A	25.7 (6.2)	350 (7.1)	419 (8.5)	1716 (34.9)	2754 (56.1)	1.58 (0.07)	56.7 (8.0)	3 (1)

Abbreviations: BF1, rural Burkina Faso data set (2017/2019/2020); BF2, rural Burkina Faso data set (2020); BF3, rural Burkina Faso data set (2019/2021); IQR, interquartile range; SD, standard deviation; WDDS-10, 10-food group Women Dietary Diversity Score.

1 Women in the sample with more than one 24-hour dietary recall.

2 Information about the pregnancy trimester was missing for 20 participants.

### Dietary diversity

**FIGURE 1:**
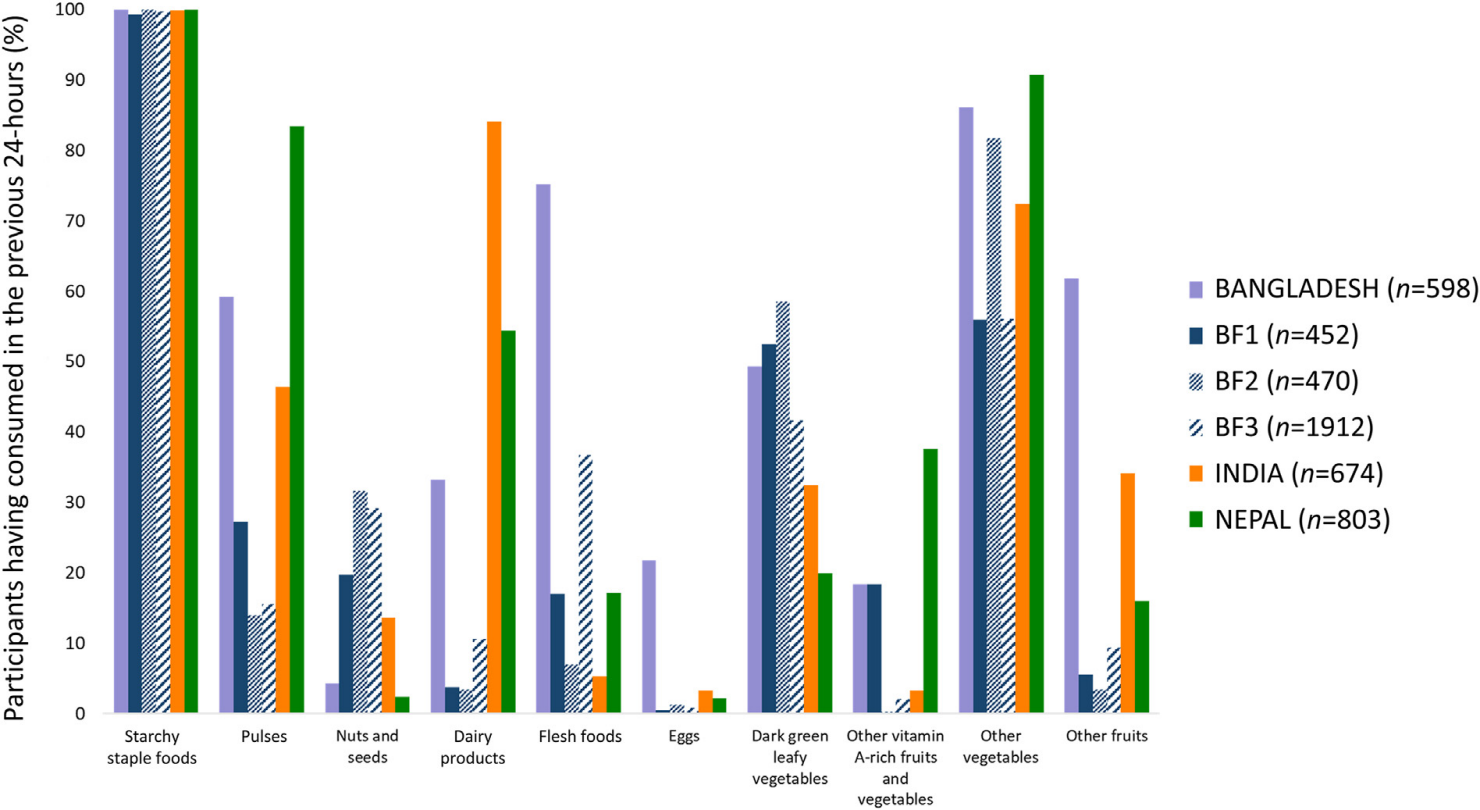
Percentage of participants having consumed the 10 food groups used to construct MDD-W in the previous 24 hours. BF1, rural Burkina Faso data set (2017/2019/2020); BF2, rural Burkina Faso data set (2020); BF3, rural Burkina Faso data set (2019/2021).

The median (interquartile range) WDDS-10 in the pooled sample was 3 (1), with higher median scores in the Bangladeshi, Nepalese, and Indian data sets compared with the 3 Burkinabé data sets (Table 1). Figure 1 shows the percentage of pregnant adolescent girls and women consuming each of the 10 food groups used to construct MDD-W across the 6 data sets. Consistently across data sets, the diet of all participants was based on starchy staple foods. Most participants consumed other vegetables but with large variations ranging from 55% in BF1 and BF3 to 91% in Nepal. The prevalence of participants consuming pulses and dairy products greatly differed across data sets: for pulses it was high in Nepal (>80%), moderate in Bangladesh and India (59 and 46%, respectively), and low in the 3 Burkinabé data sets (27% in BF1, 14% in BF2, and 15% in BF3). As for the prevalence of consumption of dairy products, it was very high in India (over 80%), moderate in Nepal and Bangladesh (53 and 33%, respectively), and low in the 3 Burkinabé data sets (4% in BF1, 3% in BF2, and 11% in BF3). In contrast, the prevalence of participants consuming nuts and seeds, and dark green leafy vegetables was higher in the 3 Burkinabé data sets. The prevalence of participants consuming flesh foods, eggs, and other fruits was higher in the Bangladeshi data sets.

### Energy and nutrient intakes and the probability of adequacy

The mean (SD) energy intake of the pregnant adolescent girls and women was 2068 (969) kcal per day in the pooled sample (Table 2), ranging from 1816 (838) kcal in BF3 to 2473 (1482) kcal in BF2. For all micronutrients apart from zinc, median intakes in the pooled sample were below the EAR (Supplemental Table 5). However, there were differences between data sets, with median intakes in the Nepalese and Bangladeshi data sets above the EAR for 5 and 4 micronutrients, respectively. Accordingly, PAs varied widely across data sets (Table 2). Across surveys, the PAs of vitamin A, riboflavin, folate, vitamin B12, calcium, and iron were <0.50. The median (IQR) MPA of the participants was 0.20 (0.34) in the pooled sample, ranging from 0.09 (0.21) in BF1 to 0.43 (0.32) in Nepal. The proportion of participants with MPA above the threshold of 0.60 was low, at 9.6% in the pooled sample and ranged from 2.4% (BF1) to 23.4% (Nepal).

**TABLE 2:** Energy intakes, probability of adequacy of individual micronutrients and mean probability of adequacy

Data set	Energy intakes, kcal/d^1^	Vitamin A^2^	Thiamin^2^	Riboflavin^2^	Niacin^2^	Vitamin B6^2^	Folate^2^	Vitamin B12^2^	Vitamin C^2^	Calcium^2^	Iron^2^	Zinc^2^	MPA^2^	MPA >0.60, *n* (%)
Bangladesh	2330 (822)	0.00 (0.60)	1.00 (0.22)	0.00 (0.25)	1.00 (0.00)	1.00 (0.00)	0.00 (0.00)	0.00 (0.07)	1.00 (0.00)	0.00 (0.00)	0.00 (0.00)	0.18 (0.70)	0.40 (0.19)	94 (15.7)
BF1	1950 (939)	0.00 (0.10)	0.00 (0.04)	0.00 (0.00)	0.00 (0.00)	0.00 (0.00)	0.00 (0.00)	0.00 (0.00)	0.00 (0.67)	0.00 (0.00)	0.00 (0.49)	0.40 (0.95)	0.09 (0.21)	11 (2.4)
BF2	2473 (1482)	0.00 (0.48)	0.00 (0.76)	0.00 (0.01)	0.00 (0.22)	0.00 (0.67)	0.00 (0.15)	0.00 (0.00)	0.01 (1.00)	0.00 (0.05)	0.00 (0.95)	0.97 (0.79)	0.16 (0.34)	69 (14.7)
BF3	1816 (838)	0.00 (0.00)	0.00 (0.01)	0.00 (0.00)	0.00 (0.03)	0.00 (0.04)	0.00 (0.00)	0.00 (0.00)	0.00 (0.05)	0.00 (0.01)	0.07 (1.00)	0.63 (0.95)	0.13 (0.21)	73 (3.8)
India	2122 (924)	0.00 (0.00)	0.80 (0.99)	0.00 (0.23)	0.20 (0.92)	0.00 (0.00)	0.00 (0.00)	0.00 (0.00)	0.04 (1.00)	0.00 (0.08)	0.00 (0.00)	0.62 (0.97)	0.20 (0.32)	35 (5.2)
Nepal	2254 (850)	0.05 (0.46)	0.96 (0.76)	0.06 (0.99)	0.64 (0.83)	1.00 (0.36)	0.00 (0.01)	0.00 (0.00)	1.00 (0.17)	0.00 (0.45)	0.00 (0.00)	0.99 (0.34)	0.43 (0.32)	188 (23.4)
Pooled	2068 (969)	0.00 (0.08)	0.03 (0.98)	0.00 (0.05)	0.03 (0.91)	0.00 (0.99)	0.00 (0.00)	0.00 (0.00)	0.02 (1.00)	0.00 (0.02)	0.00 (0.26)	0.69 (0.93)	0.20 (0.34)	470 (9.6)

Abbreviations: BF1, rural Burkina Faso data set (2017/2019/2020); BF2, rural Burkina Faso data set (2020); BF3, rural Burkina Faso data set (2019/2021); MPA, mean probability of adequacy.

1 Values are means (SD) calculated from a single 24-hour dietary recall (the first one in case of repetitions).

2 Values are medians (interquartile range).

### Association between WDDS-10 and MPA

**FIGURE 2:**
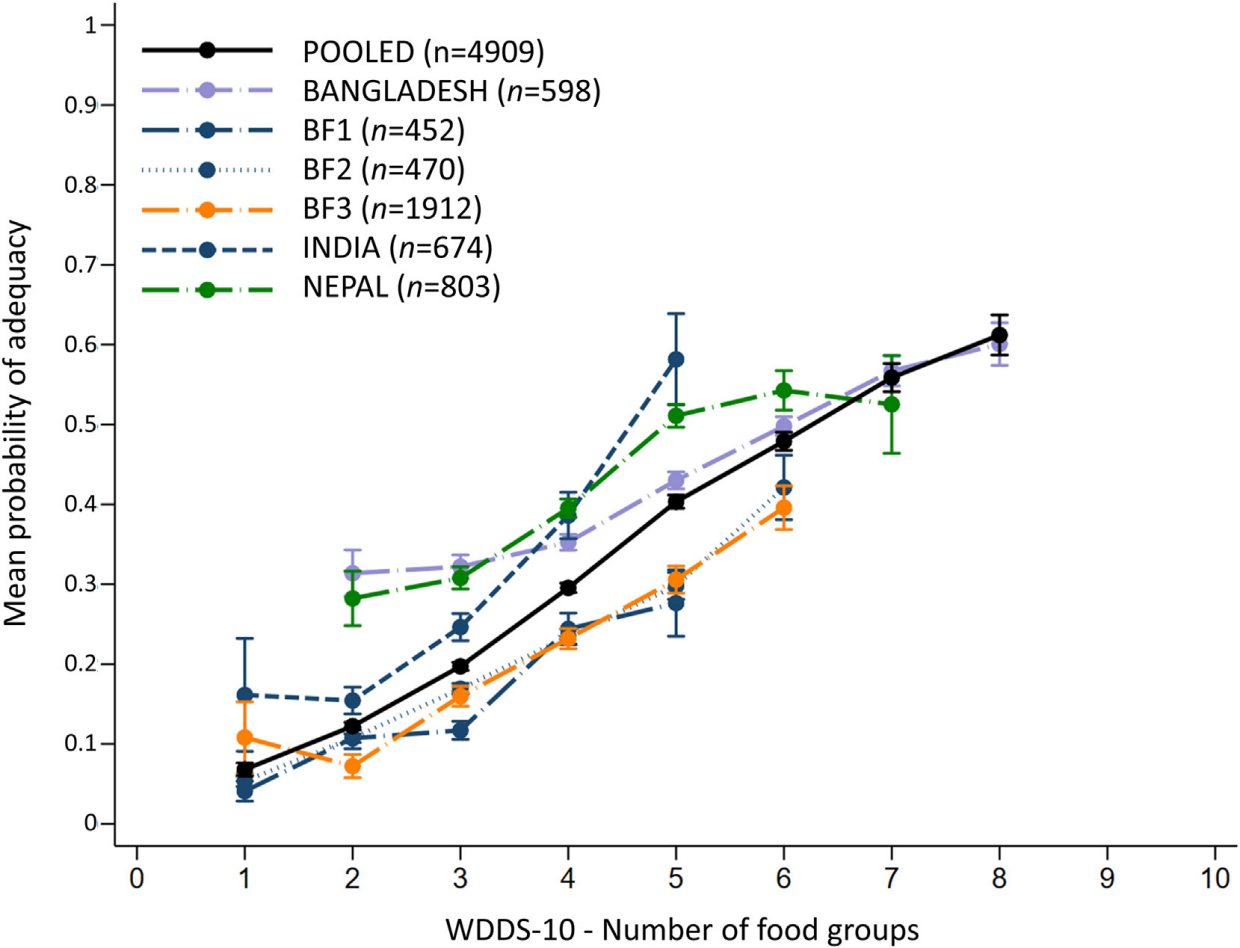
Average mean probability of adequacy by WDDS-10 score. BF1, rural Burkina Faso data set (2017/2019/2020); BF2, rural Burkina Faso data set (2020); BF3, rural Burkina Faso data set (2019/2021). Error bars represent mean ± SE. Data points representing <10 participants are not shown. Details of the number of pregnant women by data set are given in Supplemental Table 4.

Figure 2 illustrates nonadjusted associations between WDDS-10 and MPA (see Supplemental Table 6 for details of the number of pregnant women consuming various numbers of food groups by data set). The WDDS-10 was significantly and positively associated with the MPA in every data set (all *P* < 0.001) (Table 3). Unadjusted regression coefficients ranged from 0.079 (95% CI: 0.070, 0.088) to 0.309 (95% CI: 0.250, 0.367) and was 0.168 (95% CI: 0.157, 0.178) for the pooled sample. The unadjusted models explained between 14% and 33% of the MPA variance, and 28% in the pooled sample. In models including total energy intake (kcal/d) as a covariate, associations were attenuated in all data sets but remained highly significant. Energy-adjusted regression coefficients ranged from 0.038 (95% CI: 0.028, 0.050) to 0.166 (95% CI: 0.114, 0.218) and was 0.079 (95% CI: 0.069, 0.088) in the pooled sample. The energy-adjusted models explained between 29% and 66% of the MPA variance and 41% in the pooled sample.

**TABLE 3:** Linear regression of WDDS-10 with mean probability of adequacy^1^^,^^2^

Data set	Unadjusted			Total energy (kcal/d) adjusted			
	WDDS-10	Constant	Adjusted *R*^2^	WDDS-10	Energy intake, kcal/d	Constant	Adjusted *R*^2^
Bangladesh	0.079 (0.070, 0.088)	−1.06 (−1.11, −1.01)	0.333	0.055 (0.046, 0.063)	0.0001 (0.0001, 0.0001)	−1.23 (−1.27, −1.18)	0.529
BF1	0.252 (0.195, 0.310)	−2.42 (−2.60, −2.24)	0.142	0.125 (0.067, 0.183)	0.0003 (0.0003, 0.0004)	−2.65 (−2.82, −2.48)	0.291
BF2	0.309 (0.250, 0.367)	−2.20 (−2.38, −2.01)	0.185	0.166 (0.114, 0.218)	0.0002 (0.0002, 0.0003)	−2.37 (−2.52, −2.21)	0.431
BF3	0.214 (0.194, 0.233)	−2.06 (−2.13, −2.00)	0.198	0.091 (0.074, 0.108)	0.0004 (0.0003, 0.0004)	−2.40 (−2.45, −2.34)	0.488
India	0.162 (0.139, 0.186)	−1.73 (−1.83, −1.63)	0.214	0.049 (0.032, 0.067)	0.0003 (0.0003, 0.0004)	−2.00 (−2.07, −1.94)	0.662
Nepal	0.082 (0.068, 0.095)	−0.93 (−0.99, −0.87)	0.149	0.038 (0.028, 0.050)	0.0002 (0.0001, 0.0002)	−1.11 (−1.16, −1.06)	0.465
Pooled^3^	0.168 (0.157, 0.178)	−1.74 (−1.96, −1.51)	0.286	0.079 (0.069, 0.088)	0.0003 (0.0002, 0.0003)	−2.03 (−2.27, −1.78)	0.411

Abbreviations: BF1, rural Burkina Faso data set (2017/2019/2020); BF2, rural Burkina Faso data set (2020); BF3, rural Burkina Faso data set (2019/2021); WDDS-10, 10-food group Women Dietary Diversity Score.

1 Values are regression coefficients and (95% confidence intervals).

2 The mean probability of adequacy after Box–Cox transformation was used as dependent variable in all the regression models. All *P*-values are <0.001.

3 A mixed-effects regression model, including a random intercept for survey, was fitted for the pooled sample.

### Food group indicator performance and identification of thresholds

The AUC value in the pooled sample was 0.78 (95% CI: 0.75, 0.80), which indicates an acceptable predicting power, and ranged from 0.61 to 0.81 across data sets, which indicates a low to good performance in predicting an MPA > 0.60, except for BF1 where the 95% CI (0.43, 0.78) included 0.50, which indicates no statistically significant predictive power (Table 4). In the sensitivity and specificity analyses in the pooled sample, the threshold of WDDS-10 ≥5 food groups had the best performances in predicting an MPA > 0.60 (i.e., both sensitivity and specificity >0.60 and percentage of individuals correctly classified >0.70) with good sensitivity (62%), very good specificity (81%), and percentage of individuals correctly classified (79%). The threshold of ≥4 food groups showed slightly lower performances with very good sensitivity (84%) but fair enough specificity (55%) and a moderate percentage of correctly classified participants (58%). The threshold of ≥6 food groups had lower performances with low sensitivity (32%) but very good specificity (93%) and percentage of correctly classified participants (87%). The other thresholds had worse classification properties. However, findings were heterogenous across data sets. In summary, when balancing sensitivity, specificity, and percentage of correct classification, the most acceptable food group consumption threshold for predicting an MPA > 0.60 was WDDS-10 ≥ 4 in BF1, BF2, and BF3; ≥5 in India and Nepal; and ≥6 in Bangladesh.

**TABLE 4:** Test characteristics of food group indicators for classifying mean probability of adequacy >0.60 for pregnant adolescents and women^1^

Data set	AUC	WDDS-10 ≥ 4			WDDS-10 ≥ 5			WDDS-10 ≥ 6		
		Sensitivity	Specificity	PCC	Sensitivity	Specificity	PCC	Sensitivity	Specificity	PCC
Bangladesh	0.81 (95% CI: 0.77, 0.85)	98.9	19.6	32.1	97.9	41.5	50.3	78.7	67.3	69.1
BF1	0.61 (95% CI: 0.43, 0.78)	54.6	69.4	69.0	9.10	93.4	91.4	0.00	98.9	96.5
BF2	0.71 (95% CI: 0.65, 0.78)	55.1	77.3	74.0	21.7	96.8	85.7	2.9	99.8	85.5
BF3	0.74 (95% CI: 0.69, 0.79)	63.0	70.3	70.0	31.5	91.3	89.0	17.8	98.4	95.3
India	0.79 (95% CI: 0.73, 0.86)	97.1	37.7	40.8	77.1	71.5	71.8	34.3	91.6	88.6
Nepal	0.74 (95% CI: 0.71, 0.78)	94.7	30.6	45.6	70.2	70.6	70.5	26.6	92.7	77.2
Pooled	0.78 (95% CI: 0.75, 0.80)	84.0	54.9	57.7	61.7	80.6	78.8	32.1	93.2	87.4

Abbreviations: AUC, area under the curve; BF1, rural Burkina Faso data set (2017/2019/2020); BF2, rural Burkina Faso data set (2020); BF3, rural Burkina Faso data set (2019/2021); CI, confident interval; PCC, percentage correctly classified; WDDS-10, 10-food group Women Dietary Diversity Score.

1 Values are percentages (except for the AUC values).; AUC, area under the curve; CI, confident interval;

The 3 distinct scenarios from our robustness analyses returned similar findings, confirming both the observed heterogeneity across countries and also that the threshold of WDDS-10 ≥ 5 food groups had the best performance in predicting an MPA > 0.60 in the pooled sample (data not shown).

## Discussion

Following the approach used for developing and validating MDD-W among nonpregnant women [12,13], we analyzed 6 dietary data sets to determine the minimum number of food groups consumed, out of the 10 food groups of the MDD-W, which best discriminates between higher compared with lower micronutrient adequacy among pregnant adolescent girls and women in 4 LMICs. At least half of the women in each data set had PAs of 6 micronutrients at zero, highlighting the urgency of an emphasis on diet quality and nutrient adequacy population group. Consequently, pregnant adolescent girls and women had low nutrient adequacy, with median MPA values ranging from 0.09 to 0.43 across the data sets. These findings are consistent with those reported among lactating women, who also face higher nutrient requirements, where the MPA ranged from 0.23 to 0.50 in 9 data sets from resource-poor settings [12,13]. As with other population subgroups [12,13], the WDDS-10 was significantly and positively associated with MPA in each data set. Similar to the results found during the initial validation of MDD-W for nonpregnant women [12,13], our analyses showed that across the pooled sample a threshold of 5 or more food groups had the best performance in classifying pregnant adolescent girls and women as having a minimally acceptable level of dietary micronutrient adequacy (i.e., MPA > 0.60).

Nevertheless, we found evidence of heterogeneity across data sets, both in terms of dietary patterns and in the optimal threshold of WDDS-10 to predict a minimally acceptable level of micronutrient adequacy (which varied from 4 to 6). Pulses and dairy were more commonly consumed in South Asian countries, whereas nuts, seeds, and green leafy vegetables were more commonly consumed in Burkina Faso. This could be explained by geographic and temporal differences, such as food availability, prices, budgets, and preferences. For example, each data set only captured certain months of the year, whereas seasonality could affect food availability and thus dietary diversity in these contexts [39,40]. In terms of differences in thresholds, it should be noted that even in the validation study that led to adopt the MDD-W there were differences across data sets regarding the best threshold that predicted a MPA of >0.60, which varied from 4 to 6 as in the present study [12]. Various food (sub)groups contribute more or less to the MPA than others and/or can be consumed in larger or smaller quantities according to the context. This heterogeneity is not specific to pregnant adolescent girls and women. When recommending the threshold of 5 food groups, that work best in the pooled sample in this study as well as across the 9 data sets of the MDD-W validation study [12], we are pretty confident that this threshold would most likely minimize the gap to the true, context-specific, and also probably season-specific optimal threshold that remains unknown in many contexts but was found in the range of 4–6 in most if not all published studies [12,17,41].

Measuring characteristics of diets and monitoring their changes at global and national levels are needed to support governments in establishing policies and programs to promote healthy diets, assess the effectiveness of their actions, and hold them accountable. This is the spirit behind the development of the MDD-W [12,13]. Although MDD-W is already widely collected in large multitopic surveys, such as Demographic and Health Surveys and Gallup World Poll, it only reflects dietary diversity which is one, albeit indispensable, subconstruct of healthy diets [42,43]. Other promising metrics were recently designed to assess synthetically several subconstructs of healthy diets. The Global Diet Quality Score (GDQS), for example, is based on the consumption of 25 food groups that are globally important contributors to nutrient intake, on the one hand, and/or to noncommunicable disease risk, on the other hand [44]. Although it has been validated using several data sets from various contexts, the validation was performed against several outcomes and by comparisons with the performance of other metrics and not directly to nutrient adequacy. In addition, the GDQS has not yet been widely used in large surveys, probably because some appraisal of quantities or portions consumed is needed for its construction. The Global Dietary Recommendations (GDR) score is another recently developed synthetic metric that was designed to assess the adherence to a dietary pattern respecting 11 Global Dietary Recommendations from WHO, which include dietary factors protective against noncommunicable diseases [45]. Although the construction of the GDR score is based on a standardized Diet Quality Questionnaire that was validated against 24-h recalls in 3 different contexts, and has been used since in many other countries, as far as we know the GDR score itself was validated only with data from Brazil and the United States. Additional evidence is needed to establish its validity in various contexts and its equivalence across contexts [43]. Thus, MDD-W arguably remains a statically robust and valid indicator, widely collected in large multitopic surveys, to assess dietary diversity as a cornerstone of diet quality on a global and national scale. This work contributes to ongoing efforts to validate MDD-W in other populations, such as adolescents and children [43].

The present analyses have some limitations. First, despite our efforts to obtain data sets from a diversity of contexts, our study only includes data from rural contexts in 4 LMICs among 2 regions (sub-Saharan Africa and South Asia). Although our findings are not globally representative, they are consistent with other analyses among nonpregnant women from more settings [12,13]. Furthermore, the rural locations included in our study are settings where valid scores are arguably much needed because they typically have a high burden of undernutrition and low dietary diversity [15,39,46,47]. In the meantime, more data sets should be made available in settings where a reasonable proportion of pregnant adolescent girls and women reach an acceptable MPA, so that the best predictors of acceptable MPA can be further studied. For example, in the BF1 sample of our study, only 11 (2.4%) pregnant women reached an MPA ≥ 0.60, which strongly limits the search for the best dichotomous indicator predicting higher MPA. Another limitation concerns the use of an external within-person variance estimate to calculate the MPA in 4 of the 6 data sets. This results in more reliable prevalence estimates than when using a single-day recall [36], but the use of within-person variance estimates from repeated measures within the samples is preferable [35]. Although we tried to find and use an external estimate of within-person variance from a relevant food intake survey, we were limited in our ability to find studies with the same geographic (e.g., for India, the region of the external estimate study is 1500 km away from that of the data set) or temporal (different seasonality between BF1 and BF3) characteristics. Future analyses from a wider variety of settings and with data containing repeated measures are recommended to confirm that a threshold of 5 or more groups is best suited to indicate MPA > 0.60. A last limitation is the use of a set of nutrient requirements, which did not take into account the pregnancy trimester, the age of the participants or the level of bioavailability of iron and zinc. This simpler approach was preferred to take into account the fact that this information might not be accurately collected in large surveys. Nevertheless, taking these characteristics into account in 3 distinct robustness analyses did not affect our findings in terms of determining the threshold of WDDS-10 with the best classification characteristics.

In conclusion, our study suggests that the WDDS-10 is a good predictor of dietary micronutrient adequacy among pregnant adolescent girls and women in LMICs, as it was previously shown among nonpregnant and nonlactating women and lactating women [12,13]. When a dichotomous indicator is preferred over a continuous measure, our results suggest that the MDD-W may be used as a proxy indicator for higher micronutrient adequacy in LMIC contexts in all women of reproductive age, regardless of physiologic status. This might be particularly useful for international comparisons and when the physiologic status of women is unknown, which is the case in many large surveys. However, our findings suggest that context-specific thresholds might be more accurate and might, therefore, be preferred for research purposes. Given the low micronutrient adequacy in the populations studied, additional efforts are needed to enhance the diet of women of reproductive age. Although the threshold of 5 or more groups might not accurately predict micronutrient adequacy in all contexts, the indicator allows tracking processes of such efforts over time and enables benchmarking between populations. However, there is a need to provide complementary assessment of other dimensions of diet quality, such as consumption of undesired foods, food safety aspects, and within-food group contribution of foods. In addition, in food environments and diets with a considerable contribution of fortified foods, the validity of the 5 food group thresholds might require careful reconsideration.

## Author contributions

The authors’ responsibilities were as follows **–** EOV, SED, DBB, GTH-C, EL, MS, YMP, CL: designed the study; AA, EB, LD, AG, GTH-C, HH-F, SK, SSK, PHN, NMS, LMT, RRZ: provided the data sets; DBB: harmonized the data sets; SBB: analyzed data; EOV, SBB: drafted the figures, tables, and manuscript, and the other authors provided critical review; and all authors: read and approved the final manuscript.

## Conflict of interest

The authors have not conflict of interest to declare.

## Funding

This publication has been produced with the financial support of the European Commission, under the project “Knowledge and research for nutrition” implemented by Agrinatura EEIG. Its contents are the sole responsibility of the authors and do not necessarily reflect the views of the European Union and of Agrinatura EEIG.

## Data availability

Data described in the manuscript, code book, and analytic code will be made available upon request pending application and approval by the authors of the current study.
