# Supplementary material for: Defining a Dichotomous Indicator for Population-Level Assessment of Dietary Diversity Among Pregnant Adolescent Girls and Women: A Secondary Analysis of Quantitative 24-h Recalls from Rural Settings in Bangladesh, Burkina Faso, India, and Nepal
Source: Curr Dev Nutr. 2023 Nov 30;8(1):102053. doi: 10.1016/j.cdnut.2023.102053 (PMC10767136; doi:10.1016/j.cdnut.2023.102053)
Supplement: Multimedia component 1 [file mmc1.docx]

**Supplemental Methods**

## **Construction of the mean probability of adequacy**

The probability approach was used to assess nutrient adequacy. This approach is based on information or assumption about both the distribution of nutrient requirements in the population and the day-to-day variation (intra-person) in nutrient intakes. The steps required to construct the mean probability of adequacy can be summarized as follows:

1. Transform nutrient intakes using a Box-Cox transformation for energy and each micronutrient.
2. Calculate individual ($\bar{intake}_{i}$) and population ($\bar{intake}$) means of intakes for each nutrient, using the transformed variables.
3. Calculate standard deviations (SDs) and within-person variances ($\sigma_{intra}^{2}$) for the transformed intake variables.
4. Calculate inter-person variances ($\sigma_{inter}^{2}$) of transformed variables.
5. Take the number of days of 24h-recalls for each participant ($n$).
6. Compute the best linear unbiased predictor (BLUP) of the usual intake for each nutrient, for each participant, using the following equation:

| $BLUP=\bar{intake}+ \sqrt{\frac{\sigma_{inter}^{2}}{\sigma_{inter}^{2}+({\sigma_{intra}^{2}}/n)}}\times(\bar{intake}_{i}-\bar{intake})$ |
| --- |

1. Calculate the probability of adequacy for each nutrient, for each participant, based on the BLUP and transformed requirement distribution for each nutrient. This is the proportion of random values in the requirement distribution that are less than or equal to the participant's usual intake. The requirement distribution for each nutrient was simulated (n=1000) and transformed using the same power transformation as the one used for nutrient intakes.
2. Calculate the mean probability of adequacy for each participant as the mean of the probabilities of adequacy for the 11 micronutrients.

When repetitions of 24-hour dietary recalls were not available, we used the following formula to obtain an estimate of the within-person variance which takes into account the total variance of the sample:

| $\frac{external \sigma_{intra}^{2}}{{external \sigma}_{inter}^{2}}\times{internal \sigma}_{inter}^{2}$ |
| --- |

**Supplemental table 1**. Characteristics of each dataset^1^

| Dataset | Identification | Region | Location | Milieu | Objective | Dietary recall period |
| --- | --- | --- | --- | --- | --- | --- |
| Bangladesh | Pre-identified | South Asia | Northern Bangladesh | Rural | Assess the feasibility and impact of maternal nutrition packages or integrated agriculture-nutrition interventions | June - August 2015 |
| BF1 | Pre-identified | Sub-Saharan Africa | Boucle de Mouhoun, Centre-Ouest, and Haut-Bassins | Rural | Assess the feasibility and impact of maternal nutrition packages or integrated agriculture-nutrition interventions | March-June 2017, September-October 2017, September-October 2019, March-August 2020 |
| BF2 | Pre-identified | Sub-Saharan Africa | Hauts-Bassins | Rural | Assess the efficacy of fortified balanced energy-protein supplementation | September - October 2020 |
| BF3 | Pre-identified | Sub-Saharan Africa | Boucle du Mouhoun and Haut Bassins | Rural | Assess the feasibility and impact of maternal nutrition packages or integrated agriculture-nutrition interventions | November - December 2019 and January - February 2021 |
| India | Pre-identified | South Asia | Uttar Pradesh | Rural | Assess the feasibility and impact of maternal nutrition packages or integrated agriculture-nutrition interventions | 2017 and 2019 |
| Nepal | Systematic review^2^ | South Asia | Terai | Rural | Characterize the status and determinants of intra-household food and nutrient allocation, and test the effect of pregnancy interventions upon dietary intake | June - September 2015 |

^1^ BF1, rural Burkina Faso dataset (2017/2019/2020); BF2, rural Burkina Faso dataset (2020); BF3, rural Burkina Faso dataset (2019/2021). In order to identify missing data sources and assess possible selection bias of the pre-identified datasets, we ran a systematic review of studies which collected dietary intakes from pregnant women in LMICs, using one or multiple 24-hour dietary recalls. A structured search strategy, focused on title-abstract-keywords, was developed to retrieve peer-reviewed articles published in English. Searches were conducted on 31 May 2022 in Medline, Web of Science, and Scopus. The syntax of the search strategy was developed in Medline and adapted to the other two databases. All studies identified as suitable were extracted using Zotero (version 4.0.28.7). We conducted a 2-stage screening process to select the studies to be fully reviewed. During the first stage, titles and abstracts were examined by 2 authors (EOV and DBB) and irrelevant studies were excluded from further review. At the second stage, a full-text screen was performed by 2 authors (EOV and DBB) and evaluated using the following criteria for inclusion: apparently healthy pregnant women from LMICs; any study design where dietary intakes collected through one or more quantitative 24-hour recalls; associated with nutritional composition table, a sample size ≥ 100 participants; article published in English; article published in from 2010 till now. Briefly, 361 unique records were screened based on titles and abstracts and 313 irrelevant studies were excluded. Then, 48 full-texts were examined for eligibility, leading to the inclusion of 38 articles using data from 25 countries. To avoid the methodological limitations of using datasets with single quantitative 24-hour recall and the logistical difficulties of accessing external within-person variance to overcome this limitation (see the discussion section of the manuscript), our attempt to include additional datasets to our analysis focused on studies with repeated quantitative 24-hour recalls. Only 3 datasets with repeated quantitative 24-hour recalls and all the 11 micronutrients were identified and only one could be accessed.

**Supplemental table 2**. Micronutrient requirements^1^

|  | Vitamin A  (RAE), µg/d | Thiamin,  mg/d | Riboflavin,  mg/d | Niacin,  mg/d | Vitamin B6,  mg/d | Folate,  µg/d | Vitamin B12,  µg/d | Vitamin C,  mg/d | Calcium,  g/d | Iron,  g/d | Zinc,  g/d |
| --- | --- | --- | --- | --- | --- | --- | --- | --- | --- | --- | --- |
| Estimated average requirement | 370 | 1.2 | 1.2 | 14 | 1.6 | 520 | 2.2 | 46 | 800 | 24.9 | 8 |
| Standard deviation | 74 | 0.12 | 0.12 | 2.1 | 0.16 | 52 | 0.22 | 4.6 | 100 | 2.34 | 1 |

^1^ The estimated average requirement were based on the information available from WHO/FAO (28) for vitamin A, thiamin, riboflavin, niacin, vitamin B6, folate, vitamin B12, vitamin C and iron, based on the National Academy of Medicine (formerly the Institute of Medicine) for calcium (29,30) and based on the International Zinc Nutrition Consultative Group (IZiNCG) for zinc (31). The standard deviation were based on coefficient of variation from the National Academy of Medicine (29,30). All the estimated average requirements correspond to those for pregnant women in the third trimester. The estimated average requirement for iron was adjusted to 10% bioavailability (29). The estimated average requirement for zinc was for refined diets (31).

**Supplemental table 3**. Micronutrient requirements for the second robustness analysis^1^

|  | First trimester | | | | Second trimester | | | | Third trimester | | | |
| --- | --- | --- | --- | --- | --- | --- | --- | --- | --- | --- | --- | --- |
|  | Women | | Adolescent | | Women | | Adolescent | | Women | | Adolescent | |
|  | EAR | SD | EAR | SD | EAR | SD | EAR | SD | EAR | SD | EAR | SD |
| Vitamin A (RAE), µg/d | 370 | 74 | 465 | 93 | 370 | 74 | 465 | 93 | 370 | 74 | 465 | 93 |
| Thiamin, mg/d | 0,9 | 0,09 | 0,9 | 0,09 | 1,2 | 0,12 | 1,2 | 0,12 | 1,2 | 0,12 | 1,2 | 0,12 |
| Riboflavin, mg/d | 1,2 | 0,12 | 1,1 | 0,11 | 1,2 | 0,12 | 1,1 | 0,11 | 1,2 | 0,12 | 1,1 | 0,11 |
| Niacin, mg/d | 11 | 1,65 | 12 | 2 | 14,7 | 2,205 | 15,7 | 2,355 | 14,7 | 2,205 | 15,7 | 2,355 |
| Vitamin B6, mg/d | 1,7 | 0,17 | 1,6 | 0,16 | 1,7 | 0,17 | 1,6 | 0,16 | 1,7 | 0,17 | 1,6 | 0,16 |
| Folate, µg/d | 520 | 52 | 500 | 50 | 520 | 52 | 500 | 50 | 520 | 52 | 500 | 50 |
| Vitamin B12, µg/d | 2,2 | 0,22 | 2,2 | 0,22 | 2,2 | 0,22 | 2,2 | 0,22 | 2,2 | 0,22 | 2,2 | 0,22 |
| Vitamin C, mg/d | 48 | 4,8 | 43 | 4,3 | 48 | 4,8 | 43 | 4,3 | 48 | 4,8 | 43 | 4,3 |
| Calcium, mg/d | 800 | 100 | 1100 | 100 | 800 | 100 | 1100 | 100 | 800 | 100 | 1100 | 100 |
| Iron, mg/d, 5% absorption | 24 | 2,256 | 24 | 2,256 | 62,7 | 5,891 | 62,7 | 5,891 | 56 | 5,264 | 56 | 5,264 |
| Iron, mg/d, 10% absorption | 12 | 1,128 | 12 | 1,128 | 31,3 | 2,945 | 31,3 | 2,945 | 22,4 | 2,106 | 22,4 | 2,106 |
| Zinc, mg/d, 35% for refined vegetarian diets | 8 | 1 | 8 | 1 | 8 | 1 | 8 | 1 | 8 | 1 | 8 | 1 |
| Zinc, mg/d, 25% for unrefined cereal-based diets | 10 | 1,25 | 10 | 1,25 | 10 | 1,25 | 10 | 1,25 | 10 | 1,25 | 10 | 1,25 |

^1^ The estimated average requirement and standard deviation were based on the information available from WHO/FAO (28) for vitamin A, thiamin, riboflavin, niacin, vitamin B6, folate, vitamin B12, vitamin C and iron, based on the National Academy of Medicine (formerly the Institute of Medicine) for calcium (29,30) and based on the International Zinc Nutrition Consultative Group (IZiNCG) for zinc (31). The standard deviation were based on coefficient of variation from the National Academy of Medicine (29,30). The estimated average requirement for iron was adjusted to 10% bioavailability in Bangladesh, India and Nepal, and 5% in Burkina Faso (28). The estimated average requirement for zinc was adjusted to 35% bioavailability in Bangladesh, India and Nepal, and 5% in Burkina Faso (31)..

**Supplemental table 4**. Micronutrient requirements for the third robustness analysis^1^

|  | Vitamin A  (RAE), µg/d | Thiamin,  mg/d | Riboflavin,  mg/d | Niacin,  mg/d | Vitamin B6, mg/d | Folate,  µg/d | Vitamin B12, µg/d | Vitamin C, mg/d (when < 18 y) | Vitamin C, mg/d (when > 19 y) | Calcium,  g/d (when < 30 y) | Calcium,  g/d (when >31 y) | Iron,  g/d | Zinc, g/d (semi-  unrefined diet) | Zinc, g/d  (unrefined diet) |
| --- | --- | --- | --- | --- | --- | --- | --- | --- | --- | --- | --- | --- | --- | --- |
| Estimated average requirement | 540 | 1.2 | 1.5 | 14 | 1.5 | 520 | 2.2 | 75 | 80 | 750 | 860 | 22.4 | 10.2 | 11.5 |
| Standard deviation | 108 | 0.12 | 0.15 | 2.1 | 0.15 | 52 | 0.22 | 7,5 | 8 | 93 | 107 | 2.11 | 1.27 | 1.43 |

^1^ The estimated average requirement were based on the information available from by Allen et al. (32). The standard deviation were based on coefficient of variation from the National Academy of Medicine (29,30). The estimated average requirement for vitamin C and calcium took into account age. The estimated average requirement for iron was considered with low absorption for all dataset (32). The estimated average requirement for zinc was for semi-unrefined diet in Bangladesh, India and Nepal, and was for unrefined diet in Burkina Faso (32).

**Supplemental table 5**. Micronutrient intakes^1^

| Dataset | Vitamin A | Thiamin | Riboflavin | Niacin | Vitamin B6 | Folate | Vitamin B12 | Vitamin C | Calcium | Iron | Zinc |
| --- | --- | --- | --- | --- | --- | --- | --- | --- | --- | --- | --- |
| Bangladesh | 186 (397) | 1.57 (0.78) | 0.79 (0.73) | 28.4 (12.2) | 2.89 (1,32) | 228 (257) | 1.10 (1.73) | 158 (308) | 263 (384) | 8.99 (6.00) | 7.12 (3.78) |
| BF1 | 42.8 (367) | 0.70 (0.63) | 0.50 (0.59) | 5.91 (5.10) | 0.79 (0.64) | 149 (197) | 0.03 (0.14) | 12.0 (59.3) | 229 (376) | 16.9 (17.7) | 7.86 (4.70) |
| BF2 | 167 (347) | 0.82 (0,89) | 0.67 (0.56) | 8.24 (8.26) | 1.13 (1.00) | 222 (402) | 0.02 (0.20) | 35.8 (51.4) | 402 (441) | 19.1 (19.6) | 9.99 (7.29) |
| BF3 | 59.3 (138) | 0.66 (0,53) | 0.53 (0.48) | 7.17 (5.45) | 0.94 (0.74) | 154 (149) | 0.11 (0.50) | 13.7 (38.0) | 381 (326) | 21.5 (20.3) | 8.36 (5.18) |
| India | 34.7 (68.7) | 1.30 (0.85) | 0.77 (0.61) | 12.4 (8.44) | 0.60 (0.56) | 214 (150) | 0.59 (1.20) | 37.2 (46.3) | 395 (458) | 12.3 (8.65) | 8.27 (5.31) |
| Nepal | 266 (343) | 1.51 (0.99) | 1.12 (1.06) | 15.7 (9.20) | 2.09 (1.11) | 334 (223) | 0.65 (1.59) | 78.8 (146) | 552 (676) | 14.6 (8.18) | 10.9 (5.73) |
| Pooled | 92.4 (267) | 0.97 (0.97) | 0.67 (0.67) | 10.2 (11.8) | 1.14 (1.36) | 202 (215) | 0.21 (1.06) | 34.7 (80.3) | 382 (419) | 15.5 (15.1) | 8.55 (5.42) |

^1^ Values are medians (interquartile range) calculated from a single 24-hour dietary recall (the first one in case of repetitions); BF1, rural Burkina Faso dataset (2017/2019/2020); BF2, rural Burkina Faso dataset (2020); BF3, rural Burkina Faso dataset (2019/2021).

**Supplemental table 6**. Number of pregnant women per WDDS-10 score and dataset^1^

|  | WDDS-10 | | | | | | | | |
| --- | --- | --- | --- | --- | --- | --- | --- | --- | --- |
| Dataset | 1 | 2 | 3 | 4 | 5 | 6 | 7 | 8 | 9 |
| Bangladesh | 4 | 21 | 75 | 111 | 148 | 129 | 69 | 34 | 7 |
| BF1 | 22 | 133 | 156 | 111 | 25 | 5 | 0 | 0 | 0 |
| BF2 | 12 | 131 | 198 | 101 | 25 | 2 | 1 | 0 | 0 |
| BF3 | 130 | 526 | 664 | 409 | 140 | 38 | 5 | 0 | 0 |
| India | 10 | 59 | 173 | 223 | 143 | 57 | 6 | 3 | 0 |
| Nepal | 1 | 29 | 168 | 292 | 218 | 83 | 11 | 1 | 0 |
| Pooled | 179 | 899 | 1434 | 1247 | 699 | 314 | 92 | 38 | 7 |

^1^ BF1, rural Burkina Faso dataset (2017/2019/2020); BF2, rural Burkina Faso dataset (2020); BF3, rural Burkina Faso dataset (2019/2021); WDDS-10, 10-food group women dietary diversity score;
